# Longitudinal plasma nano-proteomics reveals acute systemic responses to radiotherapy and predictive biomarkers of late toxicity

**DOI:** 10.1038/s43856-026-01552-3

**Published:** 2026-04-01

**Authors:** Hanan Abumanhal-Masarweh, Salam A. Assi, Xinming Liu, Conrado Guerrero Quiles, Taha Lodhi, Kaye J. Williams, Eleanor J. Cheadle, Kostas Kostarelos, Ananya Choudhury, David C. Wedge, Catharine M. L. West, Marilena Hadjidemetriou

**Affiliations:** 1 NanoOmics Lab, Centre for Nanotechnology in Medicine, Division of Cancer Sciences, School of Medical Sciences, Faculty of Biology, Medicine and Health, The University of Manchester, Manchester, UK; 2 Wedge Group, Manchester Cancer Research Centre, The University of Manchester, Manchester, UK; 3 NIHR Manchester Biomedical Research Centre, Manchester, UK; 4 Translational Radiobiology Group, Division of Cancer Sciences and the Christie NHS Foundation Trust, Manchester Cancer Research Centre (MCRC), University of Manchester, Manchester, UK; 5 Division of Pharmacy and Optometry, Faculty of Biology, Medicine and Health, University of Manchester, Manchester, UK; 6 Targeted Therapy Group, Division of Cancer Sciences, University of Manchester, Manchester Academic Health Science Centre, Manchester, UK; 7 Nanomedicine Lab, Catalan Institute of Nanoscience and Nanotechnology (ICN2), CSIC and BIST, Campus UAB, Barcelona, Spain; 8 Institute of Neuroscience, Universitat Autònoma de Barcelona, Barcelona, Spain; 9 Institució Catalana de Recerca i Estudis Avançats (ICREA), Barcelona, Spain

**Keywords:** Tumour biomarkers, Radiotherapy

## Abstract

**Background:**

Radiotherapy induces systemic changes beyond the targeted tumour site, yet the biological mechanisms driving these effects remain poorly understood. This study aims to longitudinally profile acute systemic plasma proteomic responses to radiotherapy in patients with prostate, bladder, and head and neck cancers, providing insight into shared and tumour-specific effects and identifying biomarkers predictive of treatment-related toxicities.

**Methods:**

We apply our previously developed Nano-proteomics workflow to comprehensively analyse longitudinal weekly plasma samples collected before and during radiotherapy. We perform mass spectrometry-based differential protein analysis to identify acute changes of the plasma proteome and enriched biological pathways involved. In the prostate cohort, we associate plasma proteomics with clinical toxicity outcomes.

**Results:**

Our data indicate that the most significant systemic proteomic changes occur within the first two weeks of radiotherapy, highlighting a critical period for biomarker identification. Across all patient cohorts, we observe common biological responses: rapid activation of inflammatory and immune pathways, followed by structural reorganisation and immune resolution, regardless of tumour type or concurrent treatments. Despite this shared acute response, distinct protein mediators are found to be dysregulated in a tumour-specific manner. In the prostate cancer cohort, plasma profiling at baseline, one week after the initiation of radiotherapy, and at the end of radiotherapy,  results in the identification of 28, 29, and 20 proteins, respectively, that are associated with subsequent bowel and urinary toxicities.

**Conclusions:**

This study underscores the value of longitudinal proteomics in uncovering systemic effects of radiotherapy and supports the potential of plasma proteomic biomarkers to identify patients at increased risk of radiotherapy-induced toxicity, paving the way for personalised treatment strategies.

## Plain language summary

Radiotherapy targets tumour cells but also triggers systemic effects beyond the irradiated site. To characterise these responses, we analysed blood samples from patients with prostate, bladder, and head and neck cancers collected before and during treatment. Using a nanoparticle-enabled proteomics workflow, we profiled weekly changes in circulating proteins and observed the strongest systemic alterations within the first two weeks of radiotherapy. While many systemic responses were shared across cancer types, tumour-specific patterns also emerged. In the prostate cancer cohort, longitudinal plasma profiles revealed proteins linked to later bowel and urinary toxicities, suggesting potential predictive biomarkers. Overall, our findings underscore the systemic nature of radiotherapy and highlight longitudinal plasma proteomics as a valuable tool for biomarker discovery and personalised treatment.

## Abstract (web summary)

Abumanhal-Masarweh et al. profile plasma proteomes of cancer patients during radiotherapy and detect acute changes in inflammatory and immune pathways. Correlating these changes with toxicity data enables identification of a predictive signature for radiation-induced toxicities.

## Introduction

Radiotherapy plays a pivotal role in the management of localised solid cancers, being integrated into approximately 60% of curative-intent treatment protocols in high-income countries^1–3^. However, the heterogeneous responses of patients to radiotherapy alongside acute and chronic radiation-induced toxicities pose significant challenges to treatment outcomes and patient quality of life^4,5^.

It is now accepted that radiotherapy triggers a systemic response extending beyond its localised effect at the target tissue^2,6,7^. Despite its widespread use, the precise mechanisms governing the systemic response to radiotherapy remain largely unexplored^6,8,9^. Emerging evidence suggests that radiotherapy elicits a multifaceted response that encompasses cellular and soluble factors implicated in stress responses, inflammation, and immune reactions^2,8,9^. It is unclear whether these proteomic alterations reflect a response to tumour regression, radiation-induced toxicity, or the healing process following treatment. The systemic response to radiotherapy can interact with other anti-tumour treatments, such as chemotherapy and immunotherapy, potentially reshaping long-term outcomes and contributing to toxicities^7,9^. Understanding the underlying biological pathways governing these systemic effects might identify biomarkers to tailor personalised radiotherapy strategies^10,11^.

The majority of studies investigating the effect of radiotherapy on the plasma proteome were preclinical, with only a few clinical studies published to date^1,5,12–15^. Longitudinal studies tracking changes in the blood proteome of patients undergoing radiotherapy are sparse, impeding our understanding of the acute response to radiotherapy. Ouerhani et al. showcased the potential of plasma proteomics for the discovery of predictive biomarkers to monitor radiotherapy-induced skin injuries in brain cancer patients^5^. However, the utility of such biomarkers to guide treatment decisions remains to be fully elucidated. Furthermore, we have not yet been able to translate these proteomic changes into clinical blood tests that can detect radiation-induced toxicity early, enabling timely medical intervention or dose reduction^1,16,17^.

While mass spectrometry (MS) is a powerful tool for elucidating the systemic effects of radiotherapy, challenges persist in blood proteomic analysis^1,18–20^. These include the wide concentration range of plasma proteins and masking by highly abundant proteins like albumin^1,18–21^. Previous studies investigating the systemic impact of radiotherapy employed hypothesis-driven targeted proteomic approaches, thus limiting the discovery of novel biomarker proteins and underlying pathways involved^2,22,23^. Walker et al. demonstrated the potential of MS-based discovery proteomics in identifying predictive biomarkers to guide treatment options in non-small cell lung cancer patients undergoing radiotherapy^24^. However, most existing studies have been limited to single tumour types or cross-sectional sampling, leaving gaps in understanding the temporal dynamics and consistency of systemic proteomic responses induced by radiotherapy^12,15,25^.

To gain new insights into systemic responses to radiotherapy, further longitudinal investigations of the plasma proteome during radiotherapy and across different cancer types are required^1,18^. Integrating longitudinal profiling with multi-cohort analysis provides a unified view of how radiotherapy shapes the circulating proteome over time^1^.

Here, we conducted longitudinal plasma proteomic profiling in patients with prostate, bladder, or head and neck cancers. Leveraging our previously developed Nano-proteomics workflow^26,27^, we analysed plasma samples before and weekly during radiotherapy. Our data reveal common changes in the plasma proteome as early as one week after the initiation of radiotherapy across all cohorts. Longitudinal pathway analysis demonstrates a high degree of overlap in radiotherapy-enriched pathways among the three cancer types. Finally, correlating proteomic data with clinical toxicity outcomes in prostate cancer patients highlights the potential of baseline plasma proteomic profiling to identify biomarkers for pre-treatment prediction of radiotherapy-induced late toxicities.

## Methods

### Materials

Hydrogenated soy phosphatidylcholine (HSPC), 1,2-distearoyl-sn-glycero-3-phosphoethanolamine-N-[methoxy(polyethylene glycol)−2000 (DSPE-PEG2000) were purchased from Lipoids (USA), while cholesterol and 4-(2-Hydroxyethyl) piperazine-1-ethanesulfonic acid (HEPES) and Ammonium sulfate were purchased from Sigma (UK).

### Ethical approvals

This project was reviewed and approved by the Manchester Cancer Research Centre Biobank Sample Access Committee, and all sample collection was conducted under the MCRC Biobank Research Tissue Bank Ethics (study number 18CAWE03). Patients were consented in accordance with the Declaration of Helsinki.

### Patient cohorts and plasma samples collection

All recruited patients received treatment according to local standard-of-care protocols, with radiotherapy administered with curative intent. Patients were recruited for this study based on the following criteria: Head & Neck Squamous Cell Carcinoma (Oropharynx, T2-T4a, N2a-c, M0, receiving 66 Gy radiation in 30 fractions and cisplatin 100 mg/m^2^ or carboplatin AUC 5 or 6). Prostate (high risk patients T2c-4, N0, M0, treated with neoadjuvant hormone treatment, receiving 60 Gy radiation in 20 fractions followed by adjuvant hormones for up to 2 years). Muscle Invasive Bladder Cancer (MIBC) (T2-T3b, grade 3 receiving 52.5–55 Gy radiation in 20 fractions plus weekly Gemcitabine (100 mg/ m^2^)). Informed consent from all participants was obtained. Sample kits for blood collection were prepared by the biobank for each time point and were held in the endocrine laboratory by the research nurses. Two blood samples were collected prior to the start of radiotherapy: one at the planning appointment and one immediately before the first treatment fraction. The second pre-treatment sample was used for analysis in this study. Additional samples were collected weekly during radiotherapy for up to six weeks, with the final sample obtained on the last treatment fraction. All blood draws were performed before the patient received that day’s radiotherapy. Samples were stored at −80 °C until they were processed. Clinical information for each patient is summarised in Supplementary Data 1–3.

### Clinical outcomes scoring (prostate cancer cohort)

Late ( > 3 months) gastrointestinal (GI) and urinary toxicity (UT) in prostate cancer patients was assessed using the Common Terminology Criteria for Adverse Events (CTCAE v5) via an electronic web portal. Radiation-induced bowel toxicity (RIBT) and urinary toxicity (RIUT) were graded as follows: RIBT: Grade 1 included constipation requiring occasional stool softeners or diarrhea with <4 stools/day increase; Grade 2 included constipation requiring regular laxatives/enemas or diarrhea with 4–6 stools/day increase. RIUT: Grade 1 included occasional incontinence, microscopic hematuria, or minimal increases in frequency, urgency, dysuria, or nocturia; Grade 2 included urinary incontinence requiring pads, moderate hematuria, or moderate increases in frequency, urgency, dysuria, or nocturia. For analysis, patients were categorized into two groups: no toxicity (grade 0 for both GI and UT; *n* = 7) and late toxicity (grade 1 or 2 for GI or UT; *n* = 17). No patients had grade 3 or 4 toxicities.

### Preparation and physicochemical characterisation of liposomes

HSPC: Chol: DSPE-PEG2000 (56.3:38.2:5.5) liposomes were prepared by thin lipid film hydration method followed by extrusion using the 10 ml LIPEX® 10 Thermobarrel Extruder (Evonik Canada Inc.). The physicochemical properties of the liposome nanoparticles employed (Supplementary Fig. 1) were characterised using Zetasizer Pro instrument and automatically analysed by the ZS Xplorer software (Malvern Panalytical).

### Ex vivo incubation of plasma samples with liposomes

Liposome-plasma incubations and subsequent purifications were performed as reported previously^28–30^. In brief, 820 μL of human plasma and 180 μL of 12.5 mM PEGylated liposomes were incubated for 10 min at 37 °C, shaking at 250 rpm. Corona-coated liposomes were then separated from unbound plasma proteins by size exclusion chromatography followed by membrane ultrafiltration using Vivaspin 6 columns (10,000 MWCO, VS0602, Sartorius). For downstream analysis samples were concentrated to 100 μl. Proteins associated with recovered liposomes were quantified using the Pierce BCA protein assay kit (Thermo Fisher Scientific). Lipid concentration was quantified by Stewart assay, and protein binding (Pb) values (μg of protein/μmol of lipid) were then calculated.

### Mass spectrometry sample preparation and analysis

For mass spectrometry analysis, the S-Trap™ Micro Spin Column Digestion Protocol was applied for corona proteins digestion. Briefly, 10 μg of total protein from each sample was mixed with 10 μL of lysis buffer (50 mM TEAB with 5% SDS) and incubated at 4 °C for 1 h. The lysed samples were reduced with 5 mM dithiothreitol (DTT) and incubated at 60 °C for 10 min, followed by alkylation with 30 mM iodoacetamide (IAA) and incubation in the dark for 30 min. IAA was then quenched by the addition of DTT, and the solution was cleared by centrifugation at 14,000 × *g* for 10 min. The cleared supernatant was transferred to a fresh tube and acidified by adding phosphoric acid to a final concentration of 1.2% (w/v). S-trap binding buffer (90% methanol in 100 mM TEAB, pH 7.1) was added to increase the sample volume by six-fold. The samples were then transferred to the S-Trap™ micro spin columns (ProtiFi) and washed once with a methyl tert-butyl ether (MTBE)/methanol (10:3 v/v) solution, followed by three washes with the S-trap binding buffer. In-column digestion was performed by adding trypsin (1 μg trypsin in 20 μL) and incubating at 47 °C for 1 h. The resulting peptides were eluted and subsequently desalted in a 96-well filter plate with a 0.2 μm PVDF membrane (3504, Corning) using Oligo R3 resin beads (1-1339-03, Thermo Fisher Scientific), followed by two washes with 0.1% formic acid. Peptides were finally eluted with 0.1% formic acid in 30% acetonitrile and lyophilized using a SpeedVac vacuum concentrator (Thermo Fisher Scientific). Dried peptides were resuspended in 10 µl 0.1% formic acid in 5% acetonitrile and analysed by LC-MS/MS using an UltiMate 3000 Rapid Separation LC (RSLC, Dionex Corporation) coupled to a Q Exactive Hybrid Quadrupole-Orbitrap (Thermo Fisher Scientific) mass spectrometer. Peptide mixtures were separated using a gradient from 95% A (0.1% FA in water) and 5% B (0.1% FA in acetonitrile) to 18% B, in 34.5 min, 27% at 42.5 min, and 60% at 43.5 min, at a flow rate of 300 nL/min, using a 75 mm × 250 μm inner diameter 1.7 µM CSH C18 analytical column (Waters). Peptides were selected for fragmentation automatically by data dependant analysis (DDA). Data was acquired for 60 min in positive mode.

### Proteomics data analysis

RAW files were imported into Progenesis LC-MS software (version 3.0; Nonlinear Dynamics) with automatic feature detection enabled. A representative reference run was selected automatically to which all other runs were aligned in a pairwise manner. Automatic processing was selected to run with applied filters for peaks charge state (maximum charge 5). Protein quantitation method was selected to be the relative quantitation using Hi-N with *N* = 3 peptides to measure per protein (Hi-3), as described by Silva et al.^31^. Following peptide and protein identification, the abundance of each peptide is calculated from the whole peptide ions. For each protein, the abundance of the N most abundant peptides (*N* = 3 in our case) is averaged to provide a reading for the protein signal. This averaged reading allows relative quantitation of the same protein across runs. The resulting MS/MS peak lists were exported as a single Mascot generic file and loaded onto a local Mascot Server (Matrix Science, London, UK; version 2.5.1). The spectra were searched against the SwissProt_2018_01 and Trembl databases (selected for Homo sapiens, 161 629 entries) using the following parameters: tryptic enzyme digestion with one missed cleavage allowed, peptide charge of +2 and +3, precursor mass tolerance of 15 mmu, fragment mass tolerance of 8 ppm, oxidation of methionine (M) as variable modifications and carbamidomethyl (C) as fixed modifications, with decoy database search disabled and ESI-QUAD-TOF the selected instrument. Each search produced an XML file from Mascot, and the resulted peptides (XML files) were imported back into Progenesis LC-MS to assign peptides to features. The produced XML file was imported into Progenesis QI v3.0. Label-free quantification of plasma proteins was performed applying total ion current (TIC) normalisation. Peptide features were mapped to proteins based on peptide-spectrum matches (PSMs), retaining only PSMs with scores greater than 20 and applying a 1% false discovery rate (FDR) filter to all significant (*p* < 0.05) PSM matches. The protein abundances were log2-transformed prior to statistical analysis and samples were organised according to a within-subject design, with each individual contributing multiple samples collected across different time points. For this analysis, only patients with a complete set of samples for each specific comparison were included; therefore, no missing samples were present in the comparative analysis. Differential abundance analysis was performed using paired *t*-tests for pairwise comparisons between time points to determine the direction and magnitude of change. To correct for multiple hypothesis testing, the Benjamini–Hochberg procedure was applied, and proteins with an adjusted *p-value* < 0.05 were considered statistically significant. The resulting protein lists with one-way ANOVA *p*-value, adjusted *p-value* (*q* value), maximum fold change, and normalised protein abundance were exported from Progenesis and further analysed using Python scripts (SciPy v3.10.19). R (v. 4.2.2), and RStudio (v. 2023.06.0, PositSoftware).

### Statistical power analysis

Statistical power was calculated using a two-sided paired t-test framework based on the noncentral t distribution. For a given paired sample size (*n*), assumed effect size (*δ*, the log₂ fold change), within-subject standard deviation (*σ*), and significance level (*α*). For these analyses, σ was estimated empirically from normalised nano-proteomic data (σ ≈ 0.45 log₂ units). Effect sizes (*δ*) from 0.4–1.0 log₂ units correspond to ~1.3×–2× fold changes in protein abundance. Sample sizes were taken directly from each cohort (head & neck *n* = 11; bladder *n* = 22; prostate *n* = 26). To account for the simultaneous testing of hundreds of proteins, multiple-testing correction methods were applied. The Benjamini–Hochberg (BH) procedure was used to control the false discovery rate (FDR) at 5%. Unlike the Bonferroni method, which controls the family-wise error rate and can be overly conservative, the BH approach adaptively adjusts the significance threshold based on the rank of *p*-values, maintaining higher sensitivity. In practice, this means that for a total of *m* ≈ 400–600 proteins, the effective adjusted α (α_eff) falls between 0.001 and 0.002 when approximately 30 discoveries are expected at FDR = 5%. This value of α_eff was used in the ‘Power-BH-eff’ column as a realistic post-correction estimate. The Bonferroni correction (*α* = 0.05/m) was also evaluated as a conservative lower-bound estimate of detection power, representing a scenario where all tests are treated as independent. Together, these thresholds (*α* = 0.05, BH-adjusted, and Bonferroni) bracket the likely range of sensitivity for each cohort under different stringencies of multiple-testing control.

### Molecular pathway enrichment analysis

Pathway enrichment analysis was performed using the Enrichr web-based tool. Differentially abundant proteins (DAPs) with *p-values* < 0.05 were uploaded for each cohort and time point to investigate the biological pathways associated with radiotherapy-induced changes. Enrichment was conducted using the GO_Biological_Process_2021 gene set library. Enrichr applies Fisher’s exact test to assess over-representation, and p-values are adjusted for multiple testing using the Benjamini–Hochberg false discovery rate (FDR) method. Lists of enriched pathways, including adjusted *p*-values, the genes contributing to each pathway, odds ratios, and the number of involved proteins, were exported from Enrichr. Graphs illustrating enriched pathways across the three time points for all cohorts were generated using RStudio, including only pathways with adjusted *p-values* < 0.01.

### Clustering and kinetic plots of protein abundance

Clustering analysis was conducted on proteins showing significant changes at three key time points: 1-week post start of radiotherapy (t_1_), three weeks week post start of radiotherapy (t_3_), and at the end of radiotherapy (t_end_). The proteins were grouped into three main clusters, each reflecting distinct patterns of proteomic changes during treatment. Protein abundance for each time point was averaged across all patients, and correlations between proteins were calculated using the *corr()* function in Python. The *sch* function from the *scipy.cluster.hierarchy* library was used to cluster the correlation values with the complete linkage method and Euclidean metric. The results were visualised as dendrogram plots, and the *fcluster* function from *scipy.cluster.hierarchy* was used to define the clusters. The quality of the clustering was evaluated using the *Silhouette Score*, which quantifies the consistency and separation of the clusters. Kinetic plots were created for each defined cluster to visualise temporal trends in protein abundance across patient groups. The average abundance of each protein was calculated at each time point, and mean kinetic curves were plotted to show overall expression trends.

### Predictive modelling for toxicity

To uncover key sources of variation in the proteomic data, we analysed a cohort of 26 prostate cancer patients undergoing radiotherapy. We applied Multi-Omics Factor Analysis (MOFA), a powerful unsupervised dimensionality reduction technique designed for the integration of multi-omics datasets, including proteomics, genomics, and transcriptomics data^32^. MOFA decomposes the data into latent factors, each representing a distinct source of variance, capturing both biological and technical variability in the dataset. The *mofapy2* Python library was used to perform the MOFA analysis^33^. The optimal number of latent factors was determined using an automatic variance thresholding approach, selecting factors that explained at least 2% of the total variance. This approach ensured that the retained factors contained biologically meaningful information while minimizing overfitting, allowing for the capture of genuine biological processes^32^. After factor extraction, Hierarchical Clustering (HC) was applied to the MOFA factors to classify patients into distinct groups. The clustering was data-driven, without predefined labels, to uncover potential patient subgroups with similar patterns of proteomic variation. This unbiased approach facilitates the discovery of novel disease subtypes or treatment response profiles, which can have important implications for personalised treatment strategies in prostate cancer. To identify biomarkers associated with these latent factors, differential analysis was performed using the *ttest_ind* function from the *scipy.stats* library. To determine whether MOFA-derived patient clusters were associated with clinical toxicity outcomes, we conducted a Fisher’s exact test, a statistical method suitable for small sample sizes. This test assessed the relationship between cluster membership and RIBT/RIUT status, with a predefined significance threshold of *p* ≤ *0.05*. A statistically significant association would suggest that the proteomic-based clustering reflects clinically relevant toxicity outcomes. To identify biological mediators of radiation-induced toxicity, differential proteomic analysis was performed between the two HC-derived patient clusters. Differentially abundant proteins were identified using a two-sided t-test, with Bonferroni correction applied to control for multiple comparisons and reduce the likelihood of false positives. Proteins from Factor 1, which exhibited the highest variance between clusters, were selected for further analysis. The Bonferroni-corrected *p-value* for Factor 1 proteins was 0.028, confirming statistical significance. The top 5% of proteins with the highest absolute weights in Factor 1 were identified as the most influential features, representing potential biomarkers of radiation-induced toxicity.

### Statistical validation and permutation testing

To ensure the robustness of protein contribution weights to Factor 1, we performed permutation testing with 1000 random permutations across all proteins. This approach assessed whether the observed association between Factor 1 proteins and toxicity status was statistically significant, minimizing the risk of false-positive associations. Each permutation involved randomly shuffling the protein labels while maintaining the original sample distribution, generating a null distribution of weights against which the observed protein contributions were compared. Significance was determined based on the empirical *p*-value, calculated as the proportion of permutations where a protein’s weight exceeded the observed weight in the actual dataset. The results confirmed that the top 5% of proteins in Factor 1 remained statistically significant, with empirical *p*-values ≤ 0.05, indicating their strong association with radiation-induced toxicity outcomes. These proteins were identified as key contributors to the variance captured by Factor 1, suggesting their potential as biomarkers for radiotherapy-induced toxicity in prostate cancer patients. To further validate these findings, we conducted a differential fold-change analysis of protein abundance between toxic and non-toxic patient groups. This analysis examined whether proteins with high or low weights in Factor 1 exhibited significant changes in abundance between the two groups. Proteins identified as significant high-weight contributors in Factor 1 were found to be more than two-fold upregulated in patients who developed late radiation-induced toxicity (RIBT/RIUT) compared to those who remained toxicity-free. Conversely, low-weight contributors exhibited a greater than two-fold downregulation in the toxic patient group.

### Longitudinal toxicity predicting modelling

Plasma proteomic profiles were acquired at five serial timepoints (t_0_–t_4_). Raw MS signal intensities were log₂-transformed, normalised, and z-scored per protein to remove technical intensity variation and ensure comparability across samples. Each sample was annotated with toxicity binary outcomes, which served as clinical endpoints in all downstream analyses. Data were reformatted into long structure (sample × protein × time), ensuring compatibility with MEFISTO’s temporal modelling framework. Longitudinal proteomic measurements from the prostate cohort were modelled with MEFISTO, an extension of MOFA+ that incorporates smooth temporal structure through Gaussian process priors. The normalised proteomic matrices were supplied as a single multi-view dataset with time encoded as a continuous covariate. The model was trained with a Gaussian likelihood and variational inference. We retained 15 latent factors based on explained-variance inspection and convergence diagnostics. Outputs included: Sample-level factor values (Z) describing each patient’s latent trajectory, protein loadings (W) quantifying each protein’s contribution to each factor and temporal kernels describing the smoothness/complexity of factor evolution. These latent factors served as the basis for all subsequent association, prediction, and biomarker analyses. Statistical evaluation of factor–toxicity associations was then applied**;** for each factor ${k}$ at each timepoint $t$, factor values were compared between patients with and without late toxicity. Four complementary statistical frameworks were applied. 1. Effect size (Cohen’s d) was calculated; interpretation thresholds: 0.5 ≤ |d | <0.8: moderate and |d | ≥ 0.8: large separation. This quantified biological divergence between latent trajectories of toxicity vs non-toxicity groups. 2. Hypothesis testing (Mann–Whitney U); non-parametric two-sided U-tests evaluated distributional differences at each factor × time × toxicity combination. We report raw p-values and Benjamini–Hochberg FDR *q*-values. This identified statistically significant temporal windows of divergence between patient groups. 3. Univariable ROC–AUC screening, for each factor and timepoint, a univariable logistic model was fitted: $\mathrm{toxicity}\sim {Z}_{k}(t)$. AUC values were computed to map discriminative power across the latent space without cross-validation. Because this step is non-cross-validated, AUC values reflect maximal observable separation, not generalizable performance. 4. Bootstrap estimation of AUC uncertainty (non-nested); for each univariable AUC, a bootstrap 95% CI was computed. Resampling patients with replacement (*n* = 2000) and recomputing AUC for each resample then taking the 2.5–97.5% percentiles as the CI. This quantified variability of the signal, not generalisation.

### Nested cross-validated AUC: unbiased predictive modelling

To assess predictive performance while avoiding information leakage, we implemented a two-level nested cross-validation (CV) design, providing an unbiased estimate of model performance in a small cohort. The outer loop consisted of stratified 10-fold CV, with each fold serving as a held-out test set never used during feature selection or model training, producing the nested CV AUC as an out-of-sample performance metric. Within each outer training partition, we performed repeated stratified CV (5-fold × 50 repetitions) for feature selection and model tuning, evaluating each candidate factor independently using AUC and selecting the top-performing factors based on either a predefined threshold or ranking. Feature stability was assessed as the selection frequency across all inner CV iterations, with highly reproducible, moderately stable, and unstable factors identified based on frequency. These metrics were combined into a composite ranking score, integrating predictive strength, statistical significance, and selection stability, and a logistic regression model was trained on the selected factors to evaluate performance on the outer fold.

### Potential biomarkers evaluation: factor loadings, differential abundance, odds ratios, and AUC

For biomarker-level analysis, we focused on proteins selected from latent factors of interest (Factor 1 at t_0_, Factor 3 at t_1_, Factor 4 t_end_). For each protein, we used the corresponding MOFA/MEFISTO factor loading as a measure of its contribution to the latent factor. To quantify how extreme each loading was relative to chance, we performed a permutation test: factor loadings were randomly permuted across proteins many times (*n* ≈ 1000), and an empirical permutation p-value was computed as the proportion of permuted absolute loadings greater than or equal to the observed absolute loading. Protein-level association with late toxicity was then assessed using the log₂-normalised abundance values at the relevant timepoint. To quantify predictive strength, we fitted univariable logistic regression models with late toxicity status as the binary outcome and log₂ abundance of each protein as the predictor. From these models, we derived odds ratios (OR) per unit increase in log₂ abundance with 95% Wald confidence intervals (CI). Discrimination for each biomarker was summarised using the area under the receiver operating characteristic curve (AUC) computed from the same predictor–outcome pair. Together, these metrics (loading, permutation *p-value*, OR, and AUC) were used to identify proteins that were (i) strongly weighted on toxicity-associated latent factors, (ii) differentially abundant between toxicity groups, and (iii) individually predictive of late toxicity. (Supplementary data 17, 20–21).

### Statistics and reproducibility

Mass spectrometry data processing was performed using Progenesis LC-MS (v3.0) using default settings. Peptide-to-feature mapping was carried out using a peptide-spectrum match (PSM) score threshold > 20 and a 1% false discovery rate (FDR), ensuring high-confidence peptide assignment. Protein identification was conducted using Mascot Server (v2.5.1) against the SwissProt_2018_01 (Homo sapiens) and Trembl databases. Differential protein abundance analysis was implemented in Progenesis QI (v3.0) using a paired t-test with Benjamini–Hochberg correction (FDR < 1%). A one-way repeated-measures ANOVA was additionally applied to account for intra-patient variability across longitudinal time points. Clustering and kinetic trajectory analyses were performed in Python (SciPy v3.10.19). Hierarchical clustering was carried out using complete linkage and Euclidean distance metrics. Clusters were defined using the fcluster algorithm, and cluster robustness was assessed using the Silhouette Score. Average protein abundance per cluster was plotted over time to visualise longitudinal behaviour. Multi-Omics Factor Analysis (MOFA) was performed using mofapy2 (v1.0). Latent factors explaining ≥2% of the variance were retained. Hierarchical clustering and two-sided t-tests were used to identify key protein contributors and patient subgroups associated with each latent factor. Pathway enrichment analyses were conducted using Enrichr/GO Biological Process (2021 release) with an adjusted *p-value* threshold <0.01. Outputs included significantly enriched pathways, associated genes, odds ratios, and the number of contributing proteins.

For predictive toxicity biomarker analyses using Multi-Omics Factor Analysis (MOFA), PCA, and kinetic clustering, missing proteomic values were handled using a within-protein linear interpolation along each patient’s time course, followed by quantile normalisation across samples. Linear interpolation was applied only to intermediate missing time points (e.g., between weeks t_0_–t_end_), preserving temporal trends without altering experimentally measured endpoints. Quantile normalisation corrected for sample-level intensity differences and ensured comparable distributions across individuals and cohorts. Proteins missing across all time points were excluded from downstream analyses. After imputation, data were log₂-transformed and z-scored to minimize the influence of interpolated values on variance structure. Sensitivity assessments, including PCA structure and clustering stability, confirmed that results were robust to missingness. Interpolation primarily smoothed temporal trajectories without distorting the underlying biological patterns. Summary table of the computational methods is provided in Supplementary Data 5.

### Reporting summary

Further information on research design is available in the Nature Portfolio Reporting Summary linked to this article.

## Results

### Patient cohorts and plasma collection

**Fig. 1:**
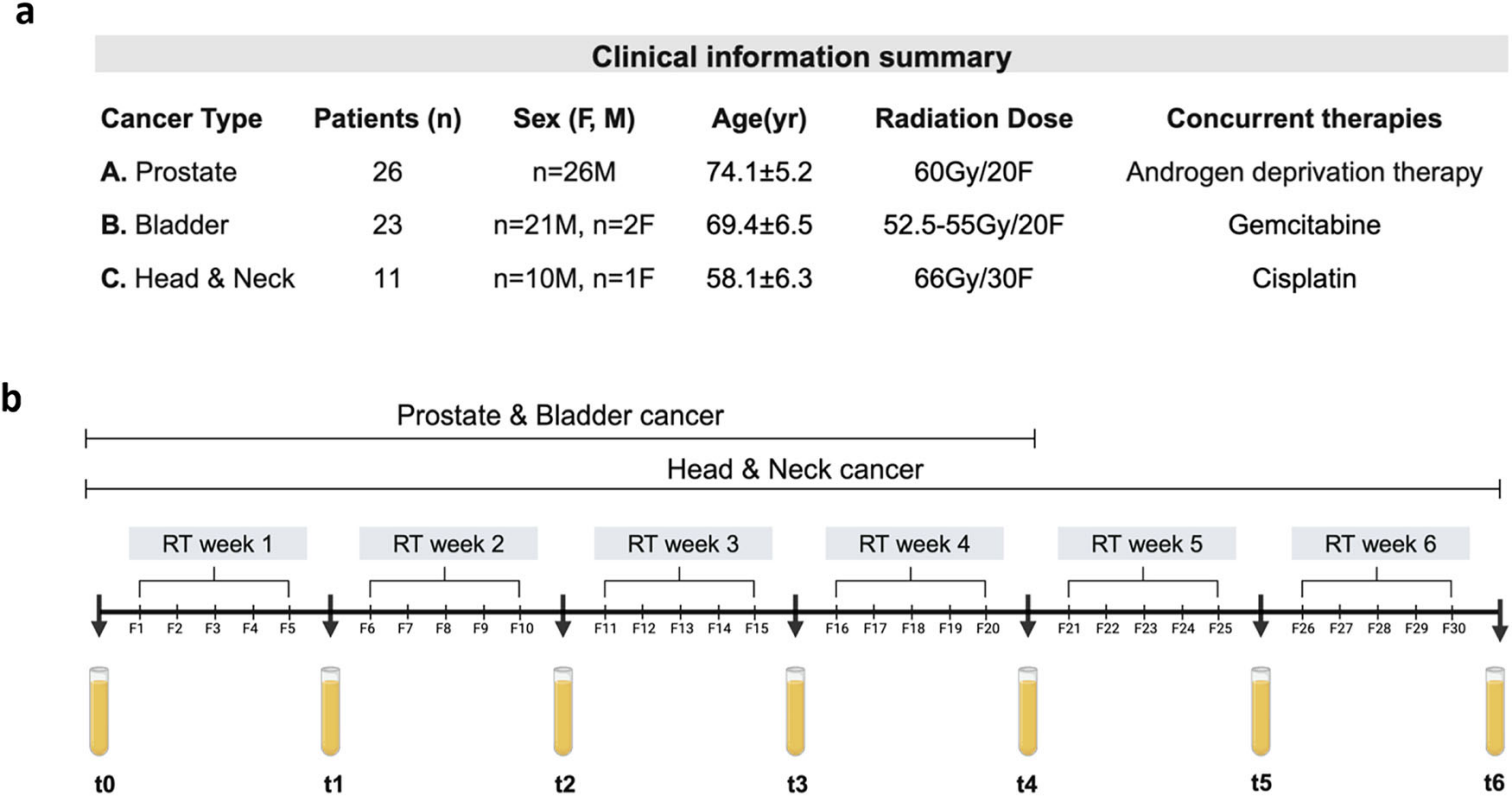
I Clinical cohorts and study design. **a** Summary of the clinical data for the three human clinical cohorts, including cancer types (prostate adenocarcinoma, urothelial carcinoma [bladder], and oropharyngeal carcinoma [head and neck]), number of patients (*n*), sex, age, total radiation dose/number of fractions, and concurrent therapies. Complete sample numbers and clinical data for each patient are provided in Supplementary Data 1–4. **b** Timeline of plasma sample collection during radiotherapy. Samples were collected each week prior to the start of radiotherapy fractions. Plasma samples were collected at baseline before radiotherapy (t₀) and weekly during radiotherapy until the end of treatment (t₁–t₄ for prostate and bladder cancers, and t₁–t₆ for head and neck cancer).

As illustrated in Fig. 1a, this study recruited cohorts of patients diagnosed with prostate adenocarcinoma (*n* = 26), bladder (urothelial) carcinoma (*n* = 23), and head and neck (oropharyngeal) carcinoma (*n* = 11). Detailed sample numbers, clinical and treatment information for each cohort are provided in Supplementary Data 1–4. To examine acute systemic changes during radiotherapy, longitudinal plasma samples were collected before (t_0_) and weekly during radiotherapy until the end of treatment (t_end_): from t_0_ to t_4_ for the prostate and bladder cancer cohorts, and from t_0_ to t_6_ for the head and neck cohort (Fig. 1b). Plasma samples were collected at each time point prior to the delivery of radiotherapy fractions.

### In-depth profiling of the plasma proteome in cancer patients undergoing radiotherapy

To conduct an in-depth analysis of the plasma proteome during radiotherapy, we employed our previously developed nanoparticle (NP)-enabled approach^26,27,34,35^. Briefly, liposome NPs were incubated ex vivo with plasma samples, allowing protein adsorption onto the NP surface, resulting in the formation of a ‘protein corona’^36,37^. Corona-coated NPs were then purified from unbound plasma proteins using size exclusion chromatography and membrane ultrafiltration. Finally, corona proteins were extracted from the NP surface and digested prior to LC-MS/MS analysis. As demonstrated by our previous work and others^29,36–38^, this workflow addresses the ‘signal-to-noise’ issue caused by the presence of albumin and other highly abundant plasma proteins. This results in the identification of low molecular weight and low-abundance plasma proteins, which are typically challenging to detect using conventional proteomics methods.

Supplementary Fig. 1a summarizes the physicochemical properties of the liposome NPs used in this study. No significant differences in protein binding were observed across different time points and patient cohorts, suggesting that radiotherapy does not quantitatively affect protein adsorption onto the NP surface (Supplementary Fig. 1b).

We first investigated whether detectable radiation-induced changes in the plasma proteome could be observed as early as 1-week post-radiotherapy. To do so, we performed differential abundance analysis comparing baseline samples (t_0_) with samples collected 1-week post-radiotherapy (t_1_) across all three patient cohorts. Given that radiotherapy-induced acute toxicities clinically manifest one to three weeks after treatment initiation^39,40^, plasma proteome profiling was also conducted 3 weeks post-radiotherapy (t_3_). Finally, to assess proteomic changes at the end of radiotherapy, comparisons were made between baseline samples and those collected after treatment completion (t_end_). Principal component analysis (PCA) of our proteomics data revealed distinct plasma proteomic profiles between the three cancer cohorts at all time points (t_0_, t_1_, t_3_, and t_end_) (Supplementary Fig. 2a).

**Fig. 2:**
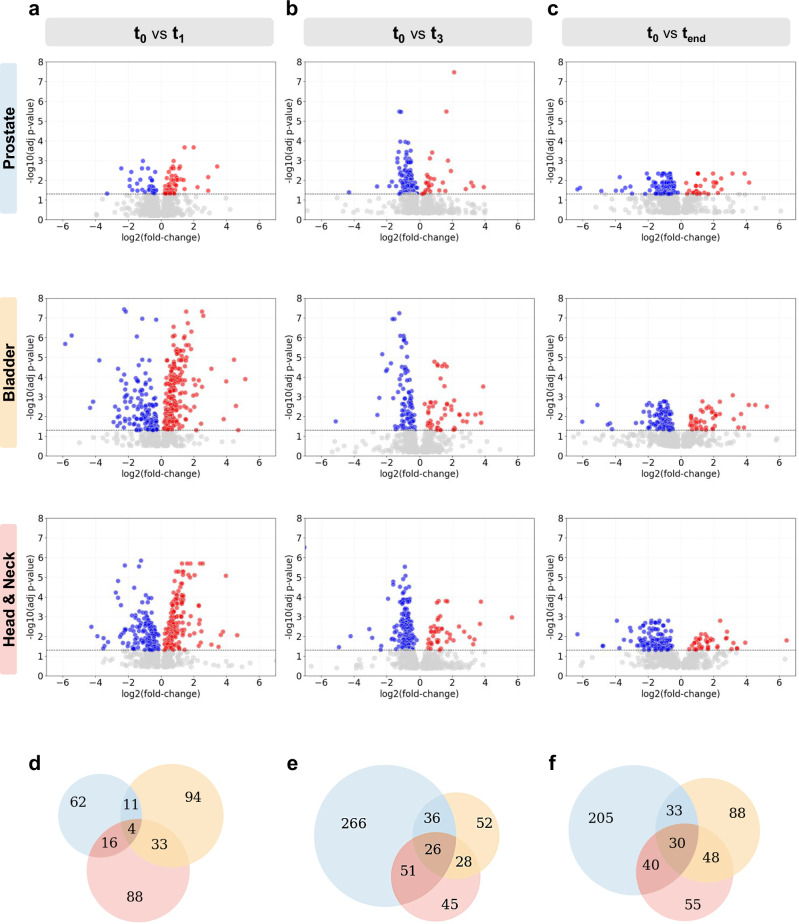
Longitudinal radiotherapy-induced plasma proteome changes. Longitudinal plasma samples from prostate, bladder, and head and neck cancer patients were analysed using the Nano-proteomics workflow. Briefly, plasma samples were incubated with liposome nanoparticles to allow protein corona formation. The corona-coated nanoparticles were subsequently recovered and purified to remove unbound proteins before protein digestion and LC-MS/MS analysis. Statistical comparisons of relative protein abundance were performed between baseline (t₀) and time points t₁ (after one week of radiotherapy), t₃ (after three weeks of radiotherapy), and t_end_ (end of radiotherapy) using Progenesis QI for Proteomics software (v3.0; Nonlinear Dynamics). **a**–**c** Volcano plots display the relationship between fold change (X-axis) and statistical significance (Y-axis) of differentially abundant proteins (DAPs) with an FDR-adjusted *p-value* (*q-value*) <0.05 at: **a** after one week of radiotherapy; **b** after three weeks of radiotherapy; and **c** at the end of radiotherapy, across the three clinical cohorts. Downregulated proteins are shown in blue and upregulated proteins in red. The total number of proteins identified per cohort and time point is provided in Supplementary Data 6 and comprehensive lists of DAPs are provided in Supplementary Data 7–9. **d**–**f** Venn diagrams illustrate the number of common and unique DAPs identified in the prostate (blue), bladder (yellow), and head and neck (red) cohorts at **d** after 1 week of radiotherapy; **e**, after three weeks of radiotherapy; and **f**, at the end of radiotherapy. Comprehensive list of common and unique DAPs across the three cohorts at each time point is provided in Supplementary Data 10.

As illustrated in Fig. 2a–c and Supplementary data 7–9, our data revealed multiple differentially abundant proteins (DAPs) with an adjusted *p-value* < 0.05 as early as 1-week post-radiotherapy: 93 in prostate cancer, 142 in bladder cancer, and 141 in head and neck cancer. Although the number of DAPs identified remained consistent across the three time points for bladder and head and neck cancer patients, an increased number of DAPs was observed in the prostate cancer cohort at both the intermediate and late stages of radiotherapy (Supplementary Fig. 2b). Additionally, while most DAPs in bladder and head and neck cancers were downregulated, the majority of DAPs in prostate cancer were upregulated (Supplementary Fig. 2b). Despite most DAPs being unique to each cohort, a total of 4, 26, and 30 proteins were commonly identified across all three cohorts at the early, intermediate, and late stages of radiotherapy, respectively (Fig. 2d–f and Supplementary data 10).

Statistical power was estimated using cohort-specific sample sizes and the observed variance in protein abundance. The prostate and bladder cohorts show strong power ( ≥ 90%) to detect moderate protein-expression changes of ~30–60% (log₂FC ≈ 0.4–0.7). The smaller head and neck cohort demonstrates only moderate power for these effects, showing ~20–30% power for changes of about 62% (log₂FC ≈ 0.7), but retains strong power ( ≥ 80%) to detect larger shifts of roughly 100% or more (log₂FC ≥ 1.0). Overall, the design provides robust sensitivity for biologically meaningful differential expression in prostate and bladder, with limited but still strong for large effects detection capability in head and neck.

### Mechanistic understanding of the systemic response to radiotherapy

**Fig. 3:**
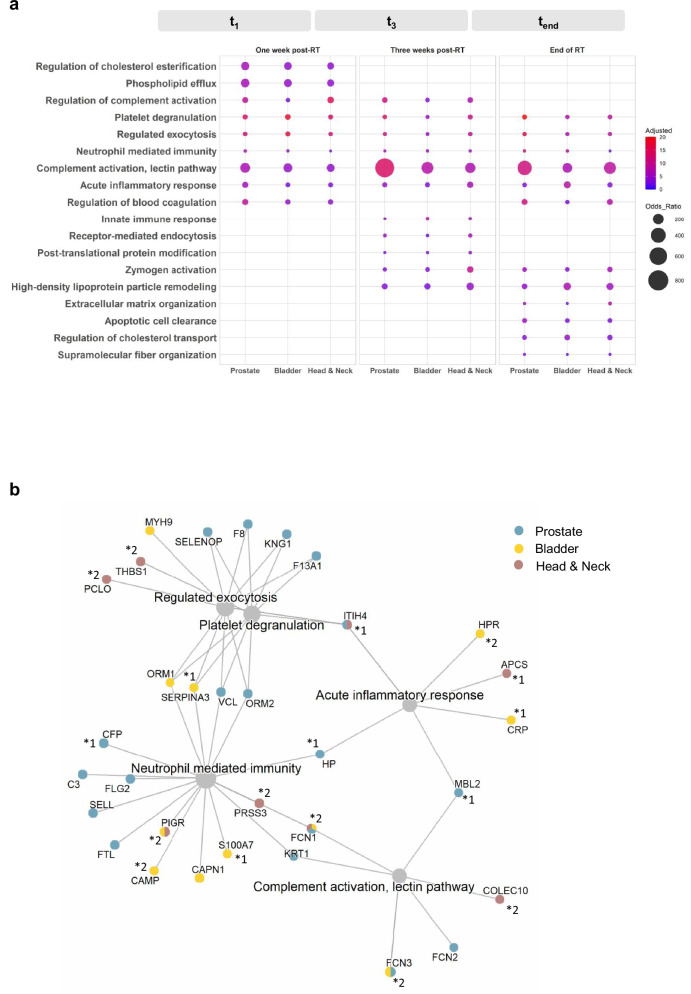
Mechanistic understanding of the radiotherapy-induced systemic response. **a** The dot plot presents the common gene ontology biological process pathways from independent enrichment analyses of each cohort. Pathways are ranked according to the adjusted *p-value* at each time point, as obtained by the Enrichr (2021) software tool for enrichment analysis. Common longitudinally enriched pathways were those consistently enriched across all time points in all three cohorts. The colour of the dots represents the adjusted *p-value*, while the size of the dots indicates the odds ratio obtained by Enrichr, reflecting the strength of association between the identified proteins and each pathway. Proteins with *p*-values < 0.05 were uploaded for each cohort and time point to investigate the biological pathways associated with radiotherapy-induced changes. A comprehensive list of these proteins is provided in Supplementary Data 7–9. Enrichment was conducted using the GO_Biological_Process_2021 gene set library (https://maayanlab.cloud/Enrichr/#libraries). Enrichr applies Fisher’s exact test to assess over-representation, and *p*-values are adjusted for multiple testing using the Benjamini–Hochberg false discovery rate (FDR) method. **b** Gene-concept network of DAPs involved in the five longitudinally common enriched pathways. Gene names encoding the proteins are used, with the colours of the gene dots representing the cohorts in which each protein was identified. Comprehensive data on the pathway involved proteins are provided in Supplementary Data 11.

To gain a mechanistic insight into the acute systemic response to radiotherapy, we performed gene ontology—biological process pathway analysis at early (t_1_), intermediate (t_3_), and end-of-treatment (t_end_) time points. As illustrated in Fig. 3a, pathway enrichment analysis revealed a dynamic response to radiotherapy, characterised by overlapping biological processes across the three cancer patients.

Following the first week of radiotherapy (t_1_), pathways related to lipid metabolism, including the regulation of cholesterol esterification and phospholipid efflux were enriched, suggesting an acute metabolic adaptation to radiation-induced oxidative stress and membrane damage (Fig. 3a and Supplementary Fig. 3a). At the intermediate time point (t_3_), the response was dominated by complement activation via the lectin pathway, along with the activation of other innate immune and vascular pathways, such as neutrophil-mediated immunity and platelet degranulation, reflecting ongoing inflammation and vascular involvement (Fig. 3a and Supplementary Fig. 3b). Finally, by the end of treatment, the molecular landscape shifted towards pathways involved in tissue remodelling and repair, including extracellular matrix organization, apoptotic cell clearance, supramolecular fibre organization, and cholesterol transport, indicating the initiation of injury-recovery processes (Fig. 3a and Supplementary Fig. 3c).

Notably, five pathways; acute inflammatory response, complement activation (lectin pathway), neutrophil-mediated immunity, platelet degranulation, and regulated exocytosis; were consistently enriched across all time points, underscoring a persistent systemic response involving immune activation and tissue damage signals (Fig. 3a). Network analysis of the *n* = 32 DAPs associated with these five pathways revealed that, although the same biological processes were enriched across all cohorts, the specific protein drivers were largely cohort specific (Fig. 3b and Supplementary data 11). This agrees with the network analysis performed for the unique for each time point enriched pathways, as illustrated in Supplementary Fig. 3a–c.

### Longitudinal monitoring of radiotherapy-induced changes in the plasma proteome

To investigate the kinetics of acute radiotherapy-induced plasma proteomic changes in each of the three cancer cohorts independently, we performed longitudinal analysis of normalised protein abundance at baseline and across all treatment time points (t_1_–t_4_ for the prostate and bladder cohorts, and t_1_–t_6_ for the head and neck cohort).

**Fig. 4:**
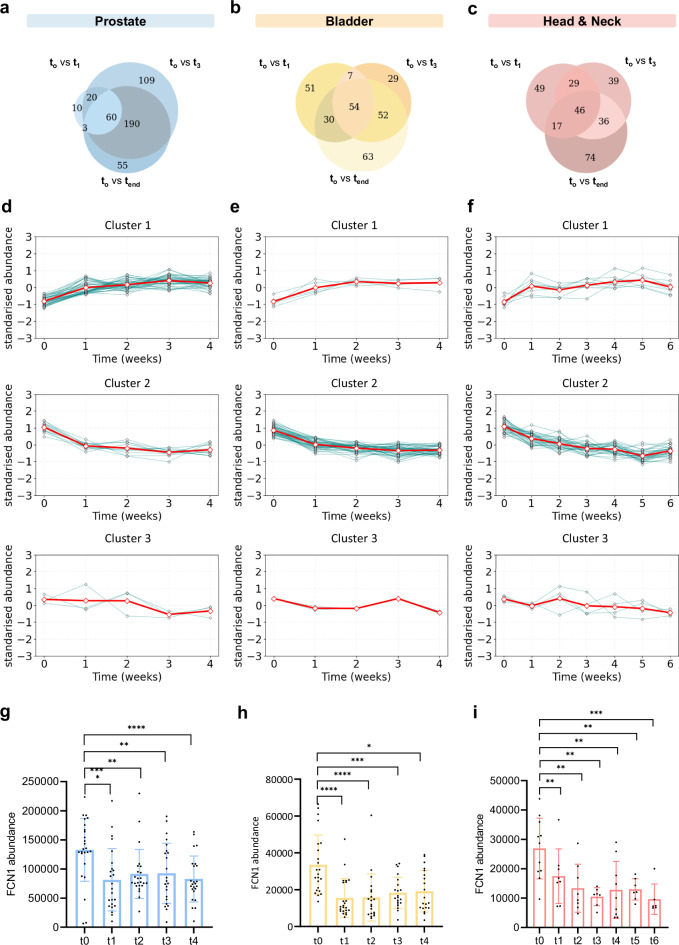
Longitudinal monitoring of radiotherapy-induced changes in the plasma proteome. The Venn diagrams display the number of common and unique DAPs identified at three time points of investigation: one-week post-radiotherapy, three weeks post-radiotherapy, and at the end of radiotherapy, for **a** prostate **b** bladder, and **c** head and neck cancer cohorts. A comprehensive list of common and unique DAPs across the three time points for each cohort is provided in Supplementary Data 12–14. **d**–**f** DAPs common between the time points were categorized into three distinct clusters using hierarchical clustering analysis for **d** prostate **e** bladder and **f** head and neck cancer patients. The Silhouette Score, used to evaluate clustering quality, is provided alongside the clustering results in Supplementary Fig. S4. **a**–**c** Full lists of proteins for each cluster per cohort are available in Supplementary Data 15. **g**–**i** The abundance values of FCN1, significantly altered during radiotherapy across all cancer cohorts, are plotted for each individual patient at the different time points (t₀–t₄ for prostate and bladder (**g**, **h**, respectively), and t₀–t₆ for head and neck (**i**). Statistical comparisons of FCN1 expression were performed using two-way ANOVA with Tukey’s multiple comparisons test between time points. Error bars represent mean ± SEM (*p-value* < 0.05 (*), *p-value* < 0.01 (**), *p-value* < 0.001 (***), *p-value* < 0.0001 (****). The exact number (*n*) of patients sampled at each time point for each cohort is provided in Supplementary Data 4. A complete list of common and unique encoded proteins found in clusters 1 and 2, identified in the prostate, bladder, and head and neck cohorts across all time points is provided in Supplementary Data 16.

As shown in Fig. 4a–c and Supplementary data 12–14, differential abundance analysis at early (t₀ vs. t₁), mid (t₀ vs. t₃), and end-of-treatment (t₀ vs. t_end_) timepoints revealed dynamic proteomic alterations over the course of radiotherapy. In the prostate cancer cohort, 60 proteins were consistently differentially abundant across all three timepoints. Similarly, 54 and 46 longitudinally shared DAPs were identified in the bladder and head and neck cohorts, respectively.

To further characterise the kinetic behaviour of the overlapping DAPs in each cohort, we performed hierarchical clustering, which revealed three major clusters with distinct abundance profiles (Fig. 4d–f, Supplementary Fig. 4d–f and Supplementary data 15). Cluster 1 included proteins with increasing abundance during treatment. Cluster 2 comprised proteins that showed sustained downregulation over time and finally, Cluster 3 included proteins with complex, non-linear kinetic profiles. While prostate cancer exhibited a predominance of proteins in Cluster 1, indicating a general trend of protein upregulation, for bladder and head and neck cancer cohorts a large proportion of DAPs fell into Cluster 2.

Among the identified radiotherapy-monitoring DAPs (Clusters 1 and 2), Ficolin-1 (FCN1), a key mediator of lectin pathway activation and neutrophil effector function, was the only common DAP showing a consistent downregulation trend across all three cohorts (Fig. 4g–i, Supplementary Fig. 4g).

### Plasma proteome profiling for predicting radiotherapy-induced toxicity in prostate cancer patients

**Fig. 5:**
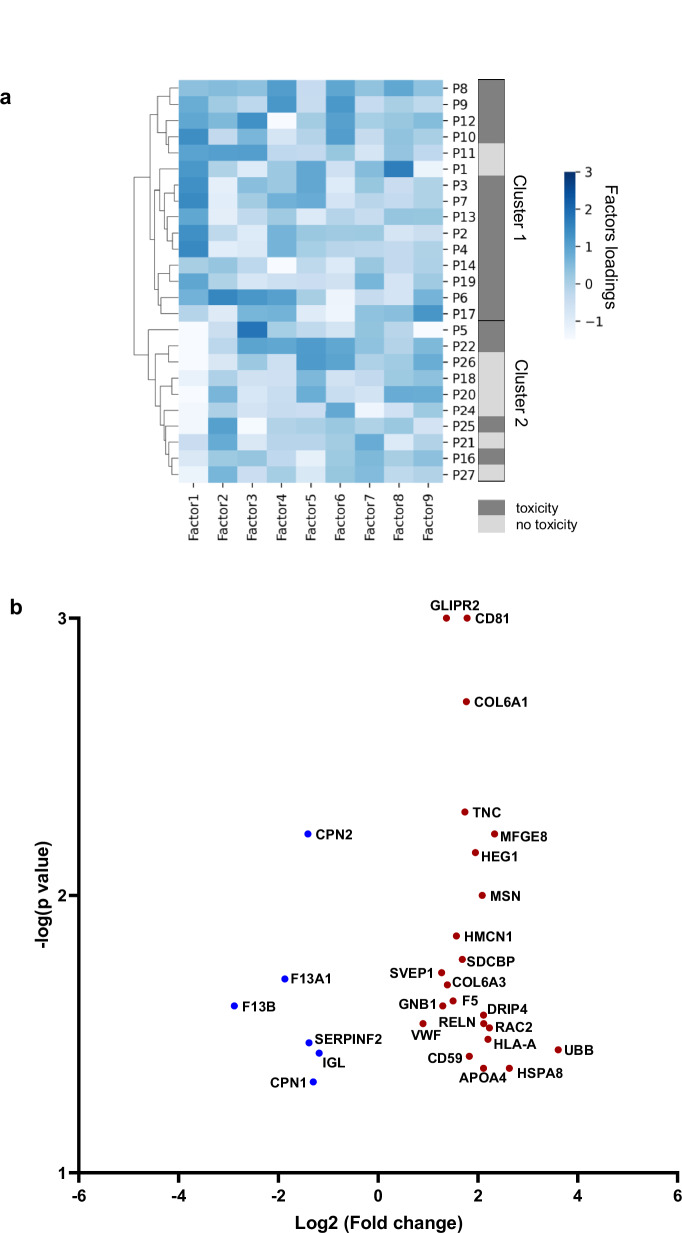
Clinical translation of radiotherapy-induced proteomic changes. **a** Heatmap of hierarchical clustering (HC) latent factors: MOFA was applied to decompose the proteomic data into latent factors explaining variance across patients without predefined labels. Only factors accounting for at least 2% of total variance were retained. HC of these factors classified patients into two distinct clusters. A two-sided Fisher’s exact test revealed a significant association between MOFA-derived clusters and clinical toxicity outcomes (the Bonferroni-corrected p-value for Factor 1 = 0.028). Patients with Radiation-Induced Bowel Toxicity (RIBT) or Radiation-Induced Urinary Toxicity (RIUT) are shown in dark grey; patients without radiation-induced toxicity are shown in light grey. **b** Volcano plot visualizing proteins within Factor 1, ranked by their relative contributions. The X-axis shows log₂ (fold change) values, and the Y-axis shows the −log₁₀ (*p*-value) obtained by two-sided permutation test. Downregulated proteins are in blue; upregulated proteins are in red. A complete list of potential biomarker proteins and associated statistics is provided in Supplementary Data 17.

To investigate whether pre-treatment plasma protein signatures can predict radiotherapy-induced toxicity, we used the prostate cancer cohort as an exemplar. We analysed baseline plasma proteomic profiles from 26 patients and examined their correlation with clinical outcomes. Of these patients, 17 developed late radiation-induced bowel (RIBT) or urinary (RIUT) toxicities more than three months after treatment, while 9 showed no signs of toxicity. To identify patterns within the proteomic data, we applied Multi-Omics Factor Analysis (MOFA). We selected the top nine factors, each explaining at least 2% of the variance, for hierarchical clustering (HC) analysis (Supplementary Fig. 5a). Factor 1 emerged as the primary driver of variation, separating patients into two distinct clusters (Fig. 5a). This clustering naturally arose from the data structure, suggesting that distinct proteomic signatures may underlie differences in patient responses to radiotherapy or their inherent risk of developing radiotherapy-induced toxicities.

To assess whether the MOFA-derived clusters were associated with toxicity outcomes, we applied Fisher’s exact test, which revealed a significant correlation between patient clusters and toxicity status (*p-value* = 0.028). To further investigate the proteomic differences underlying radiation-induced toxicity, we conducted differential proteomic analysis between the two hierarchical clustering (HC) patient clusters. Factor 1 showed a significant difference between clusters, with a Bonferroni-corrected *p*-value of 0.0029 (Supplementary Fig. 5b). Given its differential expression and correlation with toxicity, Factor 1 was selected for further analysis. From this factor, we extracted the top 5% of proteins with the highest absolute weights, representing the most influential contributors to patient clustering. Statistical significance was assessed using permutation testing with 1000 random permutations, identifying 28 candidate biomarker proteins (*p-value* ≤ 0.05) (Supplementary data 17). The volcano plot in Fig. 5b displays these potential toxicity predictive biomarkers along with their fold changes and *p-values*.

Among the 22 upregulated candidate biomarkers, several proteins involved in immune-related pathways were identified, including Tetraspanin (CD81), Complement regulatory protein (CD59), Moesin (MSN), Sushi, von Willebrand factor type A, EGF and pentraxin domain-containing protein 1 (SVEP1), Ras-related C3 botulinum toxin substrate 2 (RAC2), and HLA class I histocompatibility antigen, A-25 alpha chain (HLA-A). These proteins were enriched in pathways such as immunological synapse formation (CD81, MSN), negative regulation of complement activation (CD59, SVEP1), regulation of leukocyte migration (CD81, RAC2), and positive regulation of alpha-beta T cell proliferation and cytokine production (CD81, HLA-A), suggesting that increased immune activity and intercellular communication may predispose patients to inflammatory responses during radiotherapy. Among the six downregulated proteins, Coagulation factor XIII A chain (F13A1) and Coagulation factor XIII B chain (F13B) were significantly associated with pathways such as blood coagulation, fibrin clot formation, and the protein activation cascade, suggesting that impaired vascular and coagulation function may contribute to radiation-induced tissue damage (Supplementary data 18). Together, these observations point to a possible role of immune activation and vascular pathways in modulating the risk of radiotherapy-induced toxicity, warranting further investigation^41–43^.

To validate our findings, we implemented a full internal nested cross-validation framework, with an inner loop for feature selection and an outer loop for unbiased performance estimation. This approach minimises overfitting and prevents information leakage. We additionally performed bootstrap resampling (*n* = 2000) to quantify performance uncertainty and derive optimism-corrected AUC estimates. The nested cross-validation resulted in an AUC of 0.733 at baseline (t_0_), whereas the bootstrap analysis (1000 patient-level resamples with model refitting) produced a bootstrap AUC of 1.00 with a 95% percentile confidence interval of 0.97–1.00. These results indicate strong internal consistency of the model and robustness of the predictive signal, while highlighting the need for external validation (Supplementary Data 19).

To evaluate whether early and mid-treatment profiles (t_1_–t_3_) provide predictive information for late toxicity, and whether end-of-treatment signatures (t_end_) can identify patients at elevated risk, we used MEFISTO-derived latent factors to quantify toxicity associations and predictive performance at each timepoint independently. Associations were characterised using Cohen’s d effect sizes and AUC from univariable logistic models at each timepoint (Supplementary Fig. S6). Overall, across all latent factors and timepoints, the strongest single association was Factor 4 at t_end_, suggesting that t_end_ has toxicity predictive potential via Factor 4 (AUC = 0.93), while t_1_ shows a smaller but notable predictive signal via Factor 3 (AUC = 0.77). To identify proteins contributing most strongly to patient stratification, we extracted the top 5% of proteins with the highest absolute factor weights and *p value* < 0.05 for Factor 4 at t_end_ and Factor 3 at t_1_. Statistical testing identified 29 gene-encoded candidate biomarker proteins at t_1_ and 20 at t_end_, representing proteins with significant differential abundance that may support on-treatment monitoring or post-treatment follow-up (Supplementary Data 20–21). To evaluate the predictive value of the identified proteins at t_0_, t_1_, and t_end_, we estimated odds ratios (ORs) with 95% Wald confidence intervals (CIs) to quantify the strength and direction of association with toxicity. In addition, we assessed the discriminatory ability of each protein using the area under the receiver operating characteristic curve (AUC) (Supplementary Data 17, 20–21). Fourteen proteins at t_0_ showed AUC values > 0.7, while four proteins at t_end_ exceeded this threshold.

## Discussion

In this study, we employ our previously developed Nano-proteomics pipeline, to investigate longitudinal acute plasma proteome changes during radiotherapy in three different cancer patient cohorts. Our findings underscore a clear acute systemic effect of radiotherapy on the plasma proteome, detectable as early as 1-week post-treatment, with multiple DAPs across all cancer types. Bladder and head and neck cancers showed predominantly downregulated DAPs, whereas prostate cancer displayed mainly upregulated proteins. These differences may be attributed to the concurrent administration of radio-sensitising chemotherapy in bladder and head and neck cancer patients. Although most radiation-induced protein changes were tumour-specific, a small set of common proteins emerged at early, intermediate, and late stages, highlighting shared systemic responses to radiotherapy despite differences in treatment regimens^14^.

Investigating radiotherapy-induced plasma proteomic changes provides valuable insights into the mechanisms underlying the systemic response to treatment. Our Gene Ontology pathway analysis across early, intermediate, and end-of-treatment time points revealed a dynamic systemic response to radiotherapy, with overlapping biological processes across the three cancer cohorts. Early changes were dominated by lipid-metabolism pathways, followed by complement activation and innate immune and vascular processes at the intermediate stage, and finally pathways linked to tissue remodelling and repair at the end of treatment. Five pathways, including acute inflammatory response, complement activation, lectin pathway, neutrophil-mediated immunity, platelet degranulation, and regulated exocytosis, were consistently enriched across all time points, indicating a persistent systemic reaction. Dysregulation of similar pathways has also been reported in patients with head and neck and brain cancers undergoing radiotherapy^5,14^. Although these pathways were common across all cohorts, network analysis showed that the protein mediators driving them were largely cohort specific.

Analysis of temporal plasma protein profiles induced by radiotherapy can identify potential biomarkers for monitoring treatment outcomes and enabling more personalised therapy^9^. Longitudinal analysis of plasma proteomes using hierarchical clustering of DAPs revealed proteins with progressive increase or decrease in plasma abundance over the course of treatment, with the most dominant changes occurring within the first two weeks of radiotherapy. Among the identified radiotherapy-monitoring DAPs, FCN1 exhibited a consistent downregulation trend across all cohorts, highlighting its potential as a biological mediator of the radiotherapy response. These findings suggest that radiotherapy elicits early systemic proteomic responses, which may hold predictive value for subsequent clinical outcomes or treatment-related toxicities.

Despite the widespread use of radiotherapy in cancer treatment, clinically validated biomarkers for predicting patient susceptibility to radiation-induced toxicities remain elusive^17,44,45^. The identification of novel circulating biomarkers for monitoring radiation-induced toxicities and treatment efficacy remains a critical need in the field of radiation oncology^24,46,47^. In prostate cancer, for example, the primary treatments for localised tumours are surgery and radiation^48^. Despite dose limitations, severe gastrointestinal or genitourinary toxicity occurs in 2–6% of patients^48^. Ideal blood biomarkers could predict toxicity early in radiotherapy, before symptoms appear, allowing for treatment adjustments to prevent permanent damage. Previous studies have identified IL-2, IL-1, plasma citrulline, calprotectin, and lactoferrin as potential biomarkers of radiation-induced toxicity. However, biomarker validation data are still lacking^10,49–52^. In this study, we demonstrated that baseline and dynamic proteomic profiles during treatment are associated with the subsequent development of bowel or urinary toxicity in prostate cancer patients undergoing radiotherapy. Evaluation of AUC values identified eighteen proteins with enhanced predictive potential, with baseline biomarkers enriched in immune-related pathways. Our findings highlight the importance of integrating baseline and treatment-induced plasma proteomic changes to improve prediction of late radiation-induced toxicity. While these findings are promising, validation in larger, independent cohorts is required to establish robust predictive biomarkers. Additionally, post-treatment sampling is necessary to improve our understanding of the temporal evolution of systemic molecular responses underlying late toxicities following radiotherapy^14,15,19^.

Concurrent chemotherapy in the bladder and head and neck cancer cohorts as well as adjuvant hormonal therapy in the prostate cancer cohort, are expected to affect the systemic proteomic changes observed in our study. Despite treatment differences among the different cohorts, shared radiation-induced biological processes were identified. To strengthen the clinical utility of our findings, future studies should incorporate clinical factors, such as age, T stage, hormone therapy duration, baseline inflammatory status, comorbidities, medications, and fasting status, into multivariable models and validate the identified proteomic signatures in larger, independent, multi-centre cohorts. Such studies will be essential to confirm the predictive potential of these biomarkers and support their use in personalised radiotherapy.

## Conclusions

In this study, we longitudinally profiled 60 patients across prostate, bladder, and head and neck cancer cohorts and demonstrated that radiotherapy induces a rapid and systemic plasma proteome response. This response, detectable as early as one week into treatment, is characterised by common biological processes across cohorts that transition from early metabolic and inflammatory activity to later structural reorganization and immune resolution. Although shared pathways were enriched across tumour types, distinct protein mediators were involved in a cancer-specific manner, reflecting heterogeneity in systemic responses to radiotherapy. Collectively, our results underscore the potential of deep plasma proteomic profiling to capture early systemic effects of radiotherapy and to identify patients at increased risk of radiation-induced toxicity. By revealing proteomic signatures predictive of future toxicities, our findings suggest that pre-treatment blood-based profiling could inform early clinical decision-making, paving the way for the future development of blood biomarkers to enable personalised radiotherapy and mitigate adverse effects.

## Supplementary information

Supplementary information

Description of Additional Supplementary Files

Supplementary Dataset 1

Supplementary Dataset 2

Reporting Summary

## Data Availability

The mass spectrometry proteomics data have been deposited to the ProteomeXchange Consortium via the PRIDE [1] partner repository with the dataset identifier PXD071492 and 10.6019/PXD071492. The source data for Fig. 3(a-b), Fig. 4g–I and for the supporting Figures S1b, S2b, S3a–c, and S5a are provided in Supplementary Data set 2.

## Code availability

The underlying code for this study is available at https://github.com/assisalam/nanoOmic/ [github.com]. Computational method summary table is provided in Supplementary Data 5.
