# Supplementary material for: Longitudinal plasma nano-proteomics reveals acute systemic responses to radiotherapy and predictive biomarkers of late toxicity
Source: Commun Med (Lond). 2026 Apr 1;6:308. doi: 10.1038/s43856-026-01552-3 (PMC13212878; doi:10.1038/s43856-026-01552-3)
Supplement: Supplementary file 1 — Supplementary information [file 43856_2026_1552_MOESM1_ESM.pdf]

Longitudinal Plasma Nano-proteomics Reveals Acute Systemic Responses to Radiotherapy and Predictive Biomarkers of Late Toxicity

Hanan Abumanhal-Masarweh[1], Salam A. Assi [2,3], Xinmimg Liu[1], Conrado Guerrero Quiles[4], Taha Lodhi[4], Kaye J Williams[5], Eleanor J. Cheadle [6], Kostas Kostarelos[7,8,9], Ananya Choudhury[4], David C. Wedge[2,3], Catharine M. L. West[4], and Marilena Hadjidemetriou[1].*

[1] NanoOmics Lab, Centre for Nanotechnology in Medicine, Division of Cancer Sciences, School of Medical Sciences, Faculty of Biology, Medicine and Health, The University of Manchester, Manchester, UK.

[2] Wedge Group, Manchester Cancer Research Centre, The University of Manchester, Manchester, UK.

[3] NIHR Manchester Biomedical Research Centre, Manchester, UK.

[4] Translational Radiobiology Group, Division of Cancer Sciences and the Christie NHS Foundation Trust, Manchester Cancer Research Centre (MCRC), University of Manchester, Manchester, UK.

[5] Division of Pharmacy and Optometry, Faculty of Biology, Medicine and Health, University of Manchester, Oxford Road, Manchester, M13 9PL, UK.

[6] Targeted Therapy Group, Division of Cancer Sciences, University of Manchester, Manchester Academic Health Science Centre, UK.

[7] Nanomedicine Lab, Catalan Institute of Nanoscience and Nanotechnology (ICN2), CSIC and BIST, Campus UAB, 08193 Barcelona, Spain

[8] Institute of Neuroscience, Universitat Autònoma de Barcelona, 08913 Barcelona, Spain

[9] Institució Catalana de Recerca i Estudis Avançats (ICREA), Pg. Lluís Companys 23, Barcelona, Spain

* Correspondence should be addressed to: marilena.hadjidemetriou@manchester.ac.uk

Supplementary Figure 1

a

Lipid composition	Lipid molar ratio	Mean hydrodynamic diameter (nm)	Z-potential (mV)	Polydispersity Index (PDI)
HSPC:Chol:DSPE-PEG2000	56.3:38.2:5.5	109.1 ± 1.025	-35.09 ± 1.986	0.0302 ± 0.154

b

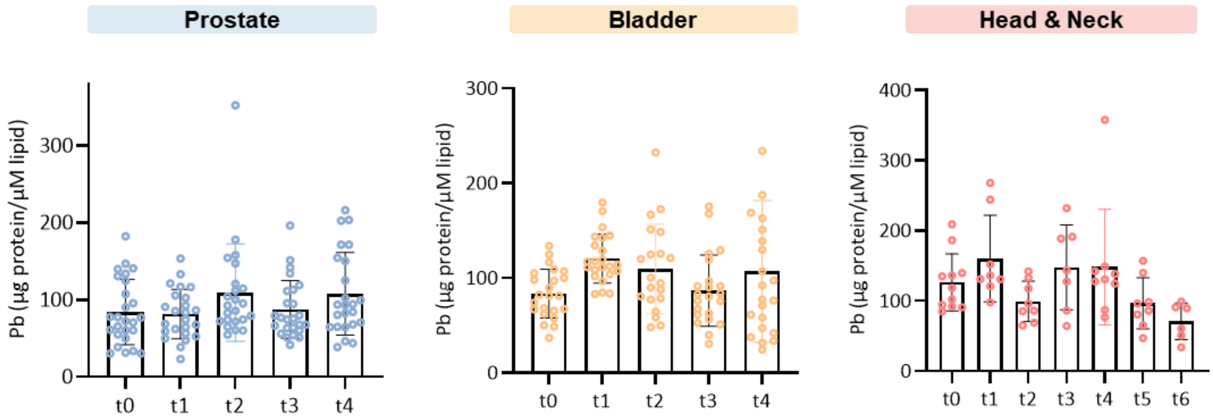

Fig.S1 I a. Physicochemical characterisation of liposome NPs employed in this study. Representative Dynamic Light Scattering (DLS) analysis showing the size (Z-average) and polydispersity index (PDI) of liposomes, along with their surface zeta potential. **b.** The total amount of protein adsorbed onto the surface of liposome NPs was quantified and expressed as protein binding value (µg of protein/µmol lipid). Error bars indicate mean ± SEM. One-way ANOVA with Tukey's multiple comparisons test between all-time points for each cancer cohort did not show significant changes in Pb values. A significant decrease in Pb values was observed only between t_1 and t_6 in the head and neck cancer cohort (* p-value = 0.0414).

Supplementary Figure 2

a

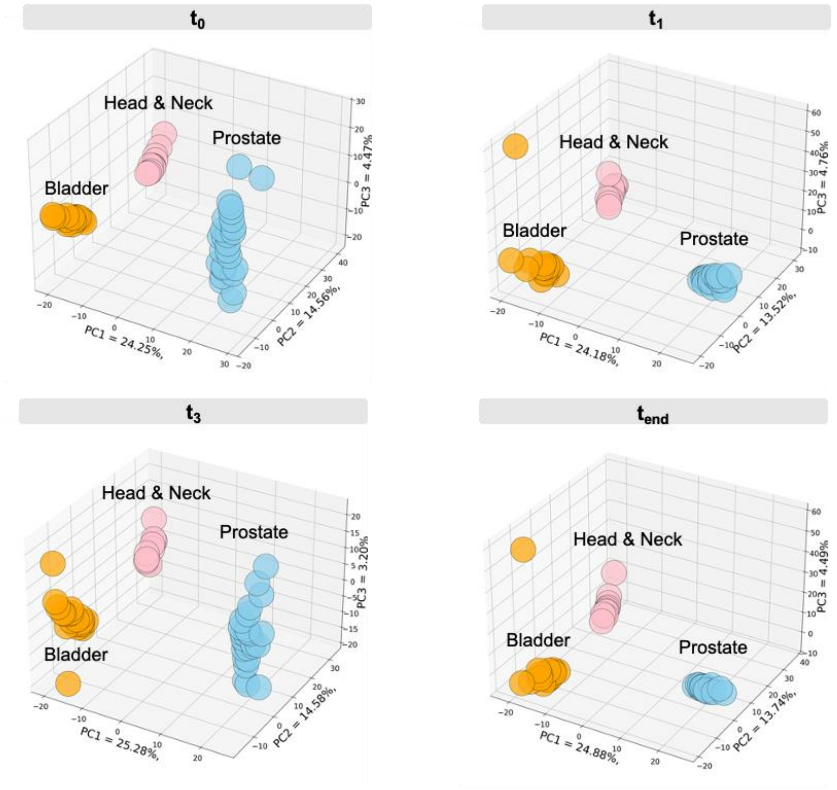

b

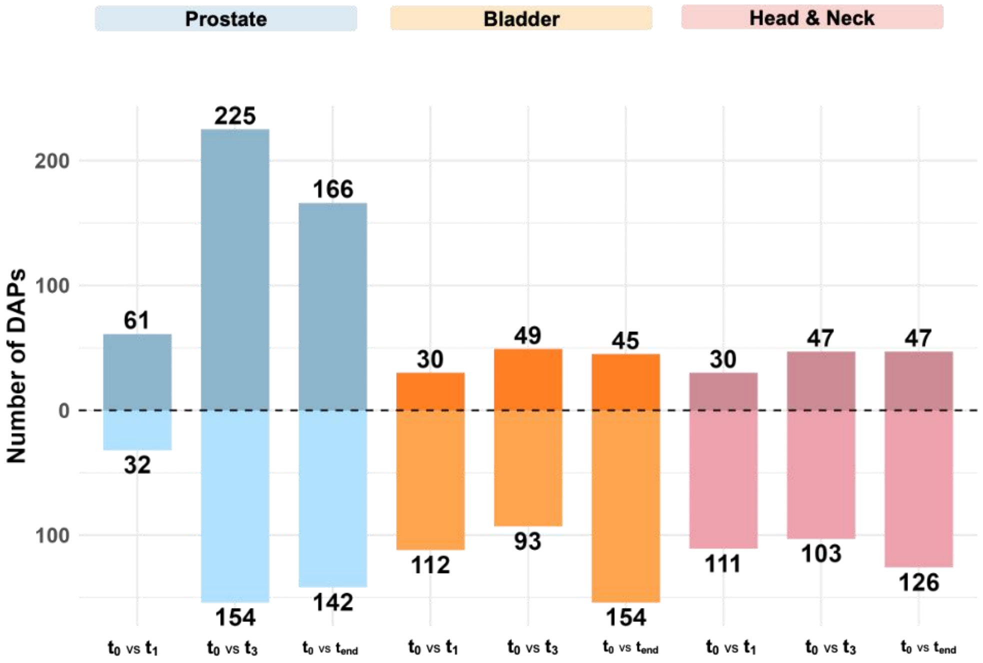

Fig.S2 I a. Principal Component Analysis (PCA) score plots showing distinct clustering of corona samples from each patient cohort, prostate, bladder, and head and neck, at baseline (t_0), early (t_1), intermediate (t_3), and end-of-treatment (t_{end}) time points. **b.** Bar graph illustrating the number of upregulated and downregulated Differentially Abundant Proteins (DAPs) identified from protein corona analysis in the three patient cohorts at each time point (t_1, t_3, t_{end}), relative to baseline (t_0).

Supplementary Figure 3

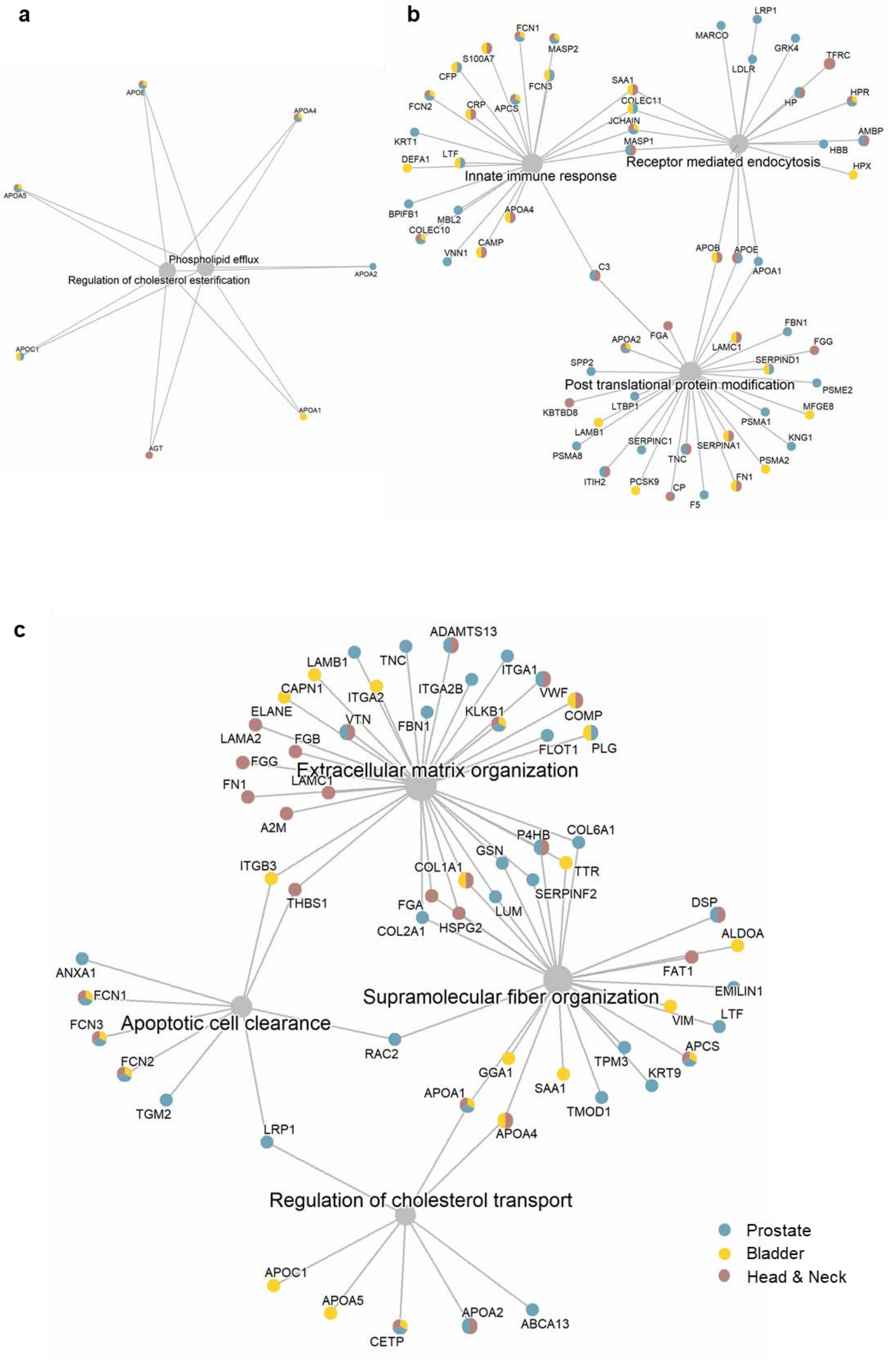

Fig.S3 I Gene-concept networks illustrating proteins involved in uniquely enriched pathways at three key time points during radiotherapy across the three clinical cohorts (prostate, bladder, and head and neck cancer): **a.** After one week of radiotherapy (t_0 vs t_1), **b.** After three weeks of radiotherapy (t_0 vs t_3), and **c.** At the end of radiotherapy (t_0 vs tend). Each concept node represents an enriched biological pathway and is sized according to the number of associated proteins. Edges connect each pathway to its respective protein nodes, which are colour-coded by cancer cohort.

Supplementary Figure 4

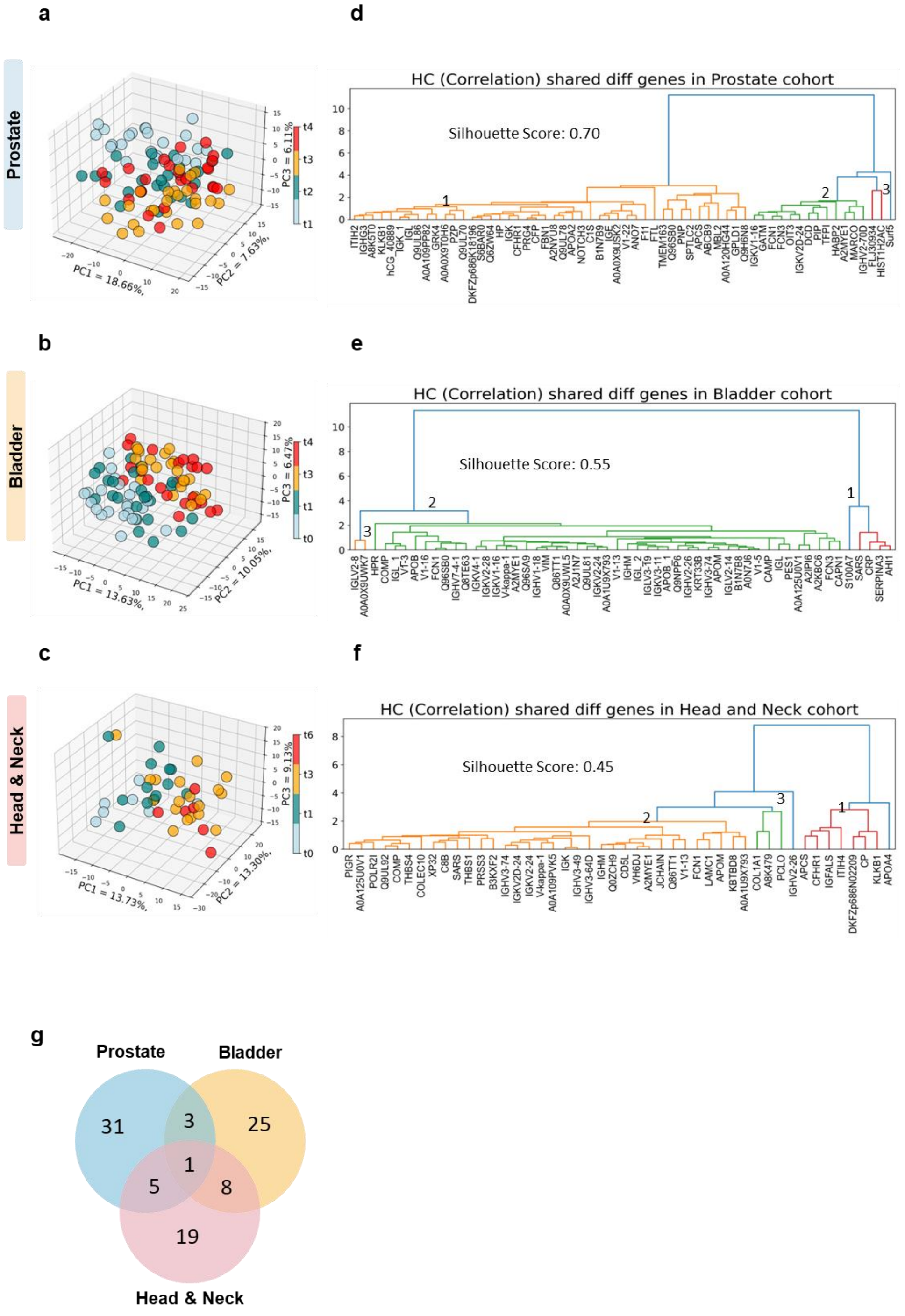

Fig.S4 I Cluster analysis of longitudinal differentially abundant proteins (DAPs) across radiotherapy time points. **a-c.** 3D PCA score plots representing the proteomic changes in each clinical cohort. Each dot corresponds to a patient, and colours indicate different time points: pre-radiotherapy (t0) and during radiotherapy at various weeks (t1, t3, t4 or t6 (t-end)). The plots illustrate the temporal shifts in the proteomic profiles for individual patients. **d–f**. DAPs identified at one week (t_1), three weeks (t_3), and end of radiotherapy (t_4) were grouped into three clusters, each representing distinct kinetic trajectories of proteomic changes. Clusters highlight unique temporal response patterns during treatment. Clustering quality was assessed using the Silhouette Score, shown alongside each result to indicate consistency and separation. **g.** Venn diagram illustrating the overlap of gene-encoded proteins in Clusters 1 and 2 across prostate, bladder, and head and neck cancer cohorts. The full list of proteins is provided in **Supplementary Data 13**.

Supplementary Figure 5

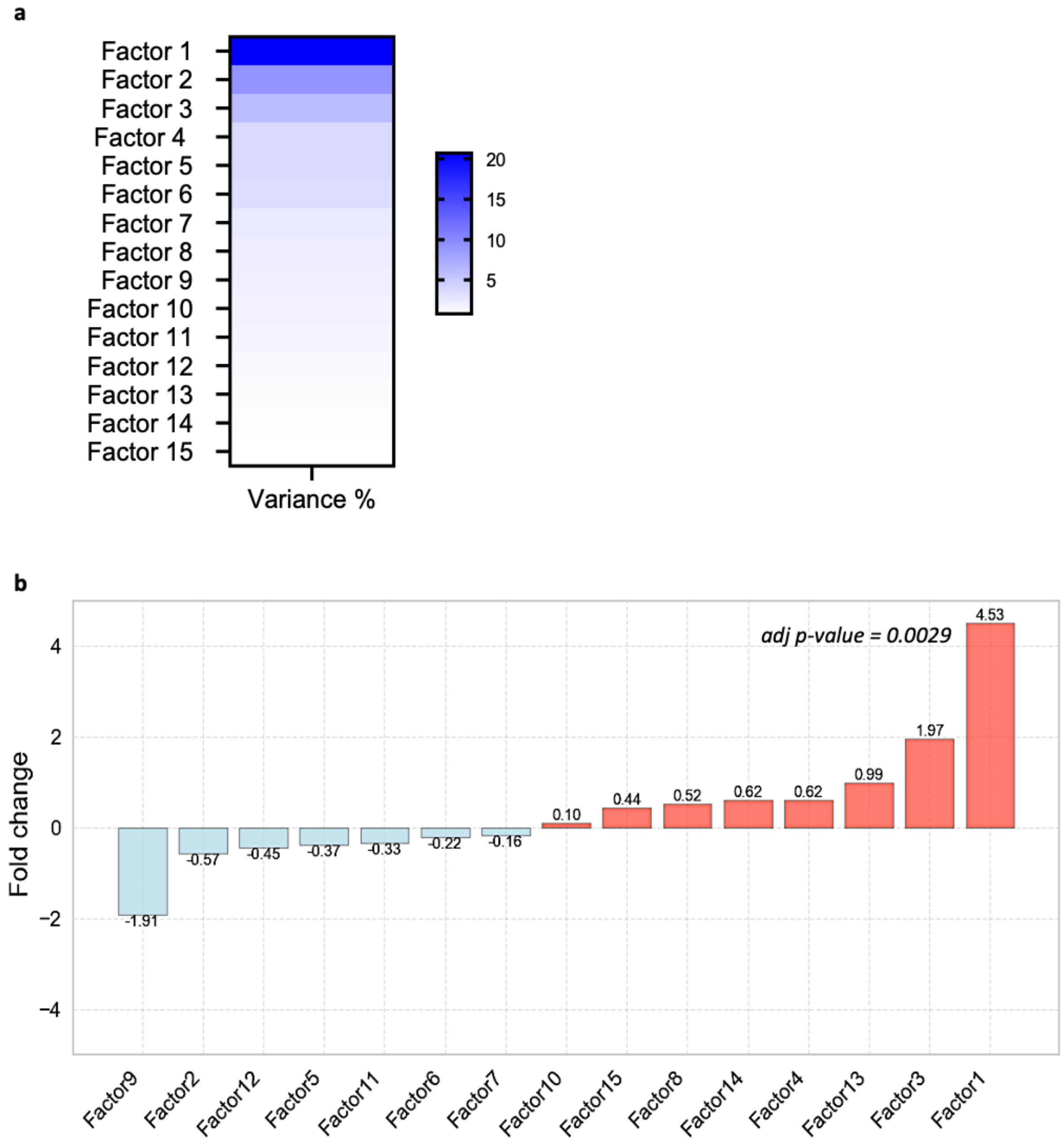

Fig.S5 I Clinical translation of radiotherapy (RT)-induced proteomic changes. **a.** Heatmap showing the proportion of variance explained by each latent factor derived from Multi-Omics Factor Analysis (MOFA). **b.** Bar plot displaying the fold change between the two hierarchical clustering (HC) patient groups (from **Fig. 5a**) across the top nine latent factors.

Supplementary Figure 6

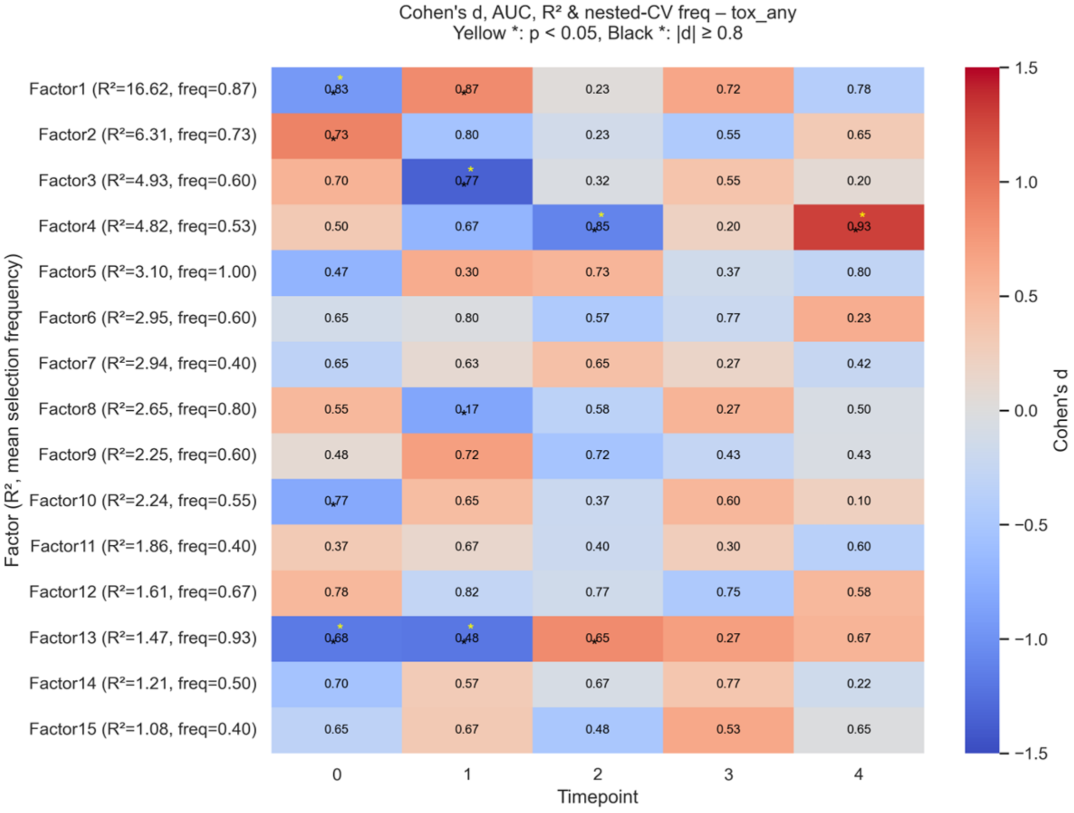

Fig. S6 I Latent Factor Associations with Late Toxicity: Heatmap summarizing the association between MEFISTO latent factors and late toxicity. Cell colours represent Cohen's d effect sizes (blue = lower in toxicity; red = higher in toxicity), while cell labels indicate the AUC of a univariable logistic model for each factor–timepoint combination. The y-axis lists factors ordered by their cross-validated R^2, along with selection frequency (freq) from nested CV, reflecting factor stability. Yellow stars mark timepoints with raw $p < 0.05$ (Mann–Whitney test), and black stars mark large effect sizes ($|d| \geq 0.8$). These annotations highlight where factor trajectories diverge between patients who later developed toxicity versus those who did not. Using a stricter threshold (raw $p < 0.05$), the toxicity signal becomes concentrated within a focused subset of factors and timepoints. Factor 4 emerged as the dominant discriminator, particularly at t_{end} (t_4), where it reached the largest effect sizes and highest AUCs (0.93). Factor 1 also demonstrated predictive relevance at baseline (t_0), with AUC > 0.8 and significant separation between toxicity and non-toxicity groups, consistent with its high R^2 and broad systemic loading pattern. A smaller but notable signal was observed for Factor 3 at t_1, although with weaker magnitude.
