# Supplementary material for: Longitudinal plasma nano-proteomics reveals acute systemic responses to radiotherapy and predictive biomarkers of late toxicity
Source: Commun Med (Lond). 2026 Apr 1;6:308. doi: 10.1038/s43856-026-01552-3 (PMC13212878; doi:10.1038/s43856-026-01552-3)
Supplement: Supplementary file 2 — Description of Additional Supplementary Files [file 43856_2026_1552_MOESM2_ESM.docx]

**Longitudinal Plasma** **Nano-Proteomics Reveals Acute Systemic Responses to Radiotherapy and Predictive Biomarkers of Late Toxicity**

Description of Additional Supplementary Files

**Supplementary Data Set 1**: This file provides the comprehensive datasets supporting the study’s primary findings.

**Contents:**

**Supplementary data 1:** Clinical information of prostate cohort patients.

**Supplementary data 2:** Clinical information of bladder cohort patients.

**Supplementary data 3:** Clinical information of head and neck cohort patients.

**Supplementary data 4:** Summary table of per-cohort sample sizes at each time point.

**Supplementary data 5:** Summary table of computational methods.

**Supplementary data 6:** Summary table of the number of identified proteins per cohort and time point.

**Supplementary data 7:** Full list of proteins identified by Progenesis analysis as differentially abundant in the prostate cohort between t_0_ and t_1_, t_0_ and t_3_, and t_0_ and t_end_. Only proteins with a p-value < 0.05 are shown. Proteins with a q-value < 0.05 were considered as DAPs in the study and included in the volcano plots in Fig. 2a–c.

**Supplementary data 8:** Full list of proteins identified by Progenesis analysis as differentially abundant in the bladder cohort between t_0_ and t_1_, t_0_ and t_3_, and t_0_ and t_end_. Only proteins with a p-value < 0.05 are shown. Proteins with a q-value < 0.05 were considered as DAPs in the study and included in the volcano plots in Fig. 2a–c.

**Supplementary data 9:** Full list of proteins identified by Progenesis analysis as differentially abundant in the head and neck cohort between t_0_ and t_1_, t_0_ and t_3_, and t_0_ and t_end_. Only proteins with a p-value < 0.05 are shown. Proteins with a q-value < 0.05 were considered as DAPs in the study and included in the volcano plots in Fig. 2a–c.

**Supplementary data 10:** Complete list of common and unique DAPs identified in the prostate, bladder, and Head & Neck cancer cohorts at the three primary time points, as illustrated in the Venn diagrams in Fig. 2d–f. The symbols ↑ (upregulated) and ↓ (downregulated) indicate the dysregulation trend of each protein.

**Supplementary data 11:** Summary table of all proteins contributing to the enrichment of common longitudinal pathways presented in Fig. 3b, including their ANOVA p-values, fold changes, dysregulation trends, and the cohorts in which they were identified across the three time points.

**Supplementary data 12:** Complete list of common and unique proteins identified across the three time points in the prostate cohort, as shown in the Venn diagrams in Fig. 4a. Cells marked with ✓ indicate the time point(s) at which each protein was detected.

**Supplementary data 13:** Complete list of common and unique proteins identified across the three time points in the bladder cohort, as shown in the Venn diagram in Fig. 4b. Cells marked with ✓ indicate the time point(s) at which each protein was detected.

**Supplementary data 14:** Complete list of common and unique proteins identified across the three time points in the head and neck cohort, as shown in the Venn diagram in Fig. 4c. Cells marked with ✓ indicate the time point(s) at which each protein was detected.

**Supplementary data 15:** Full list of common DAPs between t_0_ vs t_1_, t_0_ vs t_3_, and t_0_ vs t_end_ for the three cohorts, including the standardised abundances based on clustering analysis and the proteins in each cluster, as presented in Fig. 4d–f.

**Supplementary data 16:** List of common and unique encoded proteins found in clusters 1 and 2, identified in the prostate, bladder, and head and neck cohorts across all time points, as presented in Supplementary Fig.4g.

**Supplementary data 17:** List of 28 baseline (t_0_) DAPs contributing most strongly to MOFA weight (Factor 1), the dominant latent signature associated with late toxicity. Potential biomarkers were selected based on MOFA loading magnitude (>95th percentile), differential abundance between toxicity groups, and permutation-based loading significance. For each protein, the table reports the log2 fold change, MOFA weight (Factor 1), MEFISTO weight (Factor 1), permutation p-value for loading significance (based on 1,000 permutations), odds ratio (OR) with 95% confidence interval (CI) from univariable logistic regression, directionality (indicating whether abundance was higher or lower in the toxicity group), and AUC values, proteins with AUC > 0.7 are labelled in blue.

**Supplementary data 18:** Pathway analysis, enriched by potential toxicity-predicting biomarkers, was performed using Gene Ontology (GO) biological process analysis.

**Supplementary data 19:** Summary of nested cross-validation area under the curve (AUC) values obtained with the top identified factors, along with values obtained from bootstrap resampling.

**Supplementary data 20:** List of 29 early (t_1_) DAPs contributing most strongly to MEFISTO Factor 3, the dominant latent signature associated with late toxicity. Potential biomarkers were selected based on MEFISTO loading magnitude (>95th percentile), differential abundance between toxicity groups, and permutation-based loading significance. For each protein, the table reports the log2 fold change, MEFISTO weight (Factor 3), permutation p-value for loading significance (based on 1,000 permutations), odds ratio (OR) with 95% confidence interval (CI) from univariable logistic regression, directionality (indicating whether abundance was higher or lower in the toxicity group), and AUC values.

**Supplementary data 21:** List of 20 end-of-treatment (t_end_) DAPs contributing most strongly to MEFISTO Factor 4, the dominant latent signature associated with late toxicity. Potential biomarkers were selected based on MEFISTO loading magnitude (>95th percentile), differential abundance between toxicity groups, and permutation-based loading significance. For each protein, the table reports the log2 fold change, MEFISTO weight (Factor 4), permutation p-value for loading significance (based on 1,000 permutations), odds ratio (OR) with 95% confidence interval (CI) from univariable logistic regression, directionality (indicating whether abundance was higher or lower in the toxicity group), and AUC values, proteins with AUC > 0.7 are labelled in blue.

**Supplementary Data Set 2**: This file contains the underlying source data and raw numerical values used to generate the plots and statistical analyses for Figure 3(a-b), Figure 4g–I and for the supporting Figures S1b, S2b, S3a–c as presented in the manuscript.

**Contents:**

**Figure 3a:** Enriched pathways at different time points (t_0_ vs t_1_, t_0_ vs t_3_, and t_0_ vs t_end_), plotted using odds ratio values and −log10 adjusted p-values.

**Figure 3b:** Proteins involved in the five commonly enriched pathways across the three cohorts at the three time points.

**Figure 4(g–i):** Normalized abundance values of FCN1 for patients at all time points during radiotherapy, as obtained from Progenesis MS analysis.

**Figure S1b:** Protein binding (PB) values (μg protein/μM lipid) obtained for each plasma sample following incubation with liposomal nanoparticles.

**Figure S2**b: Number of differentially abundant proteins (DAPs) at the three time points compared with t_0_.

**Figure S3a–c:** Proteins involved in the enriched pathways at the three time points (t_0_, t_3_, and t_end_).

**Figure S5a:** Factor numbers and variance (%) values obtained from the t_0_ proteomic data.
